# Immunometabolism, Micronutrients, and Bariatric Surgery: The Use of Transcriptomics and Microbiota-Targeted Therapies

**DOI:** 10.1155/2020/8862034

**Published:** 2020-11-17

**Authors:** Shannon Galyean, Dhanashree Sawant, Andrew C. Shin

**Affiliations:** Department of Nutritional Sciences, Texas Tech University, Lubbock, Texas, USA

## Abstract

**Background:**

Obesity is associated with the gut microbiota and decreased micronutrient status. Bariatric surgery is a recommended therapy for obesity. It can positively affect the composition of the gut bacteria but also disrupt absorption of nutrients. Low levels of micronutrients can affect metabolic processes, like glycolysis, TCA cycle, and oxidative phosphorylation, that are associated with the immune system also known as immunometabolism.

**Methods:**

MEDLINE, PUBMED, and Google Scholar were searched. Articles involving gut microbiome, micronutrient deficiency, gut-targeted therapies, transcriptome analysis, micronutrient supplementation, and bariatric surgery were included.

**Results:**

Studies show that micronutrients play a pivotal role in the intestinal immune system and regulating immunometabolism. Research demonstrates that gut-targeting therapies may improve the microbiome health for bariatric surgery populations. There is limited research that examines the role of micronutrients in modulating the gut microbiota among the bariatric surgery population.

**Conclusions:**

Investigations are needed to understand the influence that micronutrient deficiencies have on the gut, particularly immunometabolism. Nutritional transcriptomics shows great potential in providing this type of analysis to develop gut-modulating therapies as well as more personalized nutrition recommendations for bariatric surgery patients.

## 1. Introduction

Obesity is a complex disease that manifests multiple dysfunctions at the level of different peripheral tissues and neural components, and studies in the last 20 years have shed much light on the mechanisms underlying the pathogenesis of obesity. One of the physiological aspects that has recently gained a lot of attention is gut microbiota (GM) and their role in nutrient metabolism. People with obesity tend to have GM with less microbial gene richness (MGR) and diversity compared to normal weight individuals. Bariatric surgery (BS), a recommended therapy for severe obesity, can dramatically change phylum, genera, and species composition of the microbiota [1]. These changes can vary; however, the most prominent changes after most types of bariatric procedures can include Firmicutes and Proteobacteria [1–3]. Common types of bariatric surgeries include Roux-en-Y gastric bypass (RYGB), sleeve gastrectomy (SG), adjustable gastric band (AGB), and biliopancreatic diversion with duodenal switch (BPD/DS) [4]. Particularly after RYGB, anatomical gut alterations that reroute the food stream bypassing most of the stomach and some of the duodenum are made that can affect the GM [1, 4, 5]. SG involves removing 80% of the stomach, whereas the BPD/DS starts like the SG but bypasses a large portion of the small intestine [4]. The AGB involves the placement of an adjustable silicone gastric band to create a smaller stomach pouch [4, 6]. The differences in the anatomical alterations can produce contrasting GM changes. There can be rapid changes that occur due to the physiological alterations from surgery alone as well as additional GM changes resulting from decreased fat mass that leads to altered microbial production of short-chain fatty acids [1–3]. Normal operations of the microbiota are dependent on the stable composition of microbes including bacteria from phyla Bacteroidetes, Firmicutes, Actinobacteria, and lesser extent of Proteobacteria. Gut dysbiosis is an imbalance in the ratio of these microbes and increase in new bacterial groups which can cause inflammatory diseases, certain autoimmune disorders, and metabolic and neurological disturbances [7].

BS can disrupt absorption of micronutrients in the gut, through reduced food intake, food intolerance, and reduced gastric secretions [8]. Micronutrient deficiencies are common after BS and can influence gut health. Low levels of micronutrients influence intestinal immune regulation and barrier function on the microbiota [9]. Therefore, therapeutic interventions like micronutrient and probiotic supplementation play an important role in restoring gut health after BS [3].

Many factors affect variability in the baseline micronutrient status including genetic makeup combined with environmental risk factor exposure and lifestyle. Understanding the role of micronutrients in the microbiome function, defining genetic factors that affect the micronutrient metabolism and interindividual variations in response to therapies for GM health, and identifying effective biomarkers of the micronutrient function may significantly contribute to developing personalized nutrition for the BS population. A review discussing genetic variants that affect micronutrient status has been published recently [10]. This narrative review aims to highlight common micronutrient deficiencies associated with BS patients and the relationship between micronutrients and GM function, particularly immunometabolism. In addition, current therapies for modulating gut health and the role of nutrigenomics for patients having BS will be examined.

## 2. Methods

The authors searched for studies in the areas of micronutrient deficiencies in BS, effect of micronutrient deficiency on GM,GM-targeted therapies, and use of transcriptome analysis in BS and non-BS populations. The databases including PubMed, Medline, and Google Scholar were searched from 1976 to 2020 to obtain eligible studies. Key terms such as gut microbiome, micronutrient deficiency, gut-targeted therapies, transcriptome analysis, micronutrient supplementation, and bariatric surgery were used. Furthermore, information from review articles was included to define concepts and relevant mechanisms. Additionally, the review articles were examined to obtain related research articles.

### 2.1. Inclusion/Exclusion Criteria

Studies considered eligible for this review were (1) peer-reviewed articles, (2) articles published or available in English, (3) articles evaluating the effect of BS on GM, (4) articles related to diet, micronutrients and GM, (5) articles that included use of transcriptomics in BS population, (6) studies using gut-targeted therapies/interventions in BS patients and healthy population, (7) studies showing effects of micronutrient supplementation on gene expression, and (8) studies using transcriptomic analysis to evaluate the effect of probiotic/prebiotic supplementation. Since a limited number of studies were available in BS populations using gut therapies and transcriptome analysis, studies in non-BS patients were also included in this review. Due to the limited number of human subject studies, in vitro and animal studies related to transcriptomics and effects of probiotic/prebiotic supplementation were also included. All the authors confirmed that the articles were appropriate and eligible for inclusion in the review.

## 3. Results

We selected 104 relevant research articles, which fulfilled our inclusion criteria. Of these, 22 studies were conducted in the US, and the remaining were conducted in other countries. Of the articles included, 29 were published between 1985 and 2010, and 75 were published between 2011 and 2020. We divided the articles into the following categories to organize our results: (1) studies showing effect of diet and micronutrients on GM (31 articles); (2) studies related to diet, micronutrients, and GM in BS (18 articles); (3) studies related to diet, micronutrients, and immunity (14 articles); (4) studies showing effect of gut-targeted therapies (12 articles); (5) studies showing use of gut-targeted therapies in BS patients (5 articles); (6) studies showing use of gut-targeted therapies in non-BS patients (6 articles); (7) Studies showing use of transcriptome analysis in BS patients (4 articles); (8) studies showing effects of micronutrient supplementation on the gene expression in non-BS populations (7 articles); and (9) studies that used transcriptome analysis to evaluate effects of probiotic/prebiotic supplementation (7 articles).

### 3.1. Altered Gut Microbiota Related to Bariatric Surgery and Diet

Many studies have demonstrated substantial alterations in the composition of the GM following BS [1].  Table 1 presents common bacterial species and their role in human health [11–18]. Prominent alterations in intestinal bacteria postsurgery were increased and decreased Proteobacteria (depends on surgery type), increased Bacteroidetes, and decreased Firmicute*s* [19–23]. Other reports include an increase in Verrucomicrobia and Fusobacteria and decrease in Actinobacteria [5, 24]. Differences in composition can be due to the differences after malabsorptive procedures and exhibit a stronger effect on microbial composition compared to purely restrictive procedures [3].

Additionally, research has shown that diet composition affects GM [25]. One aspect of diet-related changes to the microbiota in BS patients is calorie restriction. Calorie restriction can be as high as 50% of the preoperative caloric consumption for the first 6 months postsurgery [26, 27]. Calorie restriction significantly alters the microbiota composition by increasing Bacteroidetes and decreasing Actinobacteria [28, 29]. Altogether, these studies provide clear evidence that BS and the consequent dietary changes, and nutritional status can dramatically shift GM diversity and compositions conducive to metabolic benefits.

### 3.2. Micronutrients and Their Effects on the Gut Microbiome

The potential for micronutrient deficiencies is present both before and after bariatric surgery. Many patients with obesity consume diets of excess calories but low in nutrient-dense foods such as fruits and vegetables. Increased adiposity can also disrupt fat-soluble vitamin absorption with rates of vitamin D deficiency averaging 60% among surgery candidates prior to surgery [30–32]. Depending on the type of surgery, patients can have additional problems postop with adequate intake, adherence to micronutrient supplementation recommendations, and malabsorption of micronutrients that exacerbate the deficiencies or establish new ones [31, 33, 34]. Micronutrient deficiencies can be a long-term issue for many patients after bariatric surgery and have a wide array of symptoms. Prevalence of deficiencies can range from 10 to 74%, with anemia being the most common [8, 35, 36]. Because micronutrients act as cofactors/coenzymes in metabolism, gene regulators, and antioxidants, low levels can cause deleterious effects involving DNA synthesis, gene expression, and oxidative stress [37]. Studies reveal that metabolic processes, such as glycolysis, TCA cycle, and oxidative phosphorylation, as well as free fatty acid synthesis and oxidation, are associated with the innate and adaptive immune systems, also known as immunometabolism [38, 39]. Associations have been made between obesity and inflammation that promote metabolic and immunologic abnormalities and can lead to many chronic issues like type 2 diabetes, cardiovascular diseases, cancer, and neurodegeneration. There is evidence of a role involving memory T cells in the TCA cycle for ATP synthesis and CD8^+^ effector T cells as well as lipopolysaccharide (LPS)-activated M1 macrophages in glycolysis [40–42]. Balancing immunometabolism is complicated due to variations in GM composition among individuals, as well as dietary impact on both the composition and function of GM [43–46]. Investigations into the influence that micronutrient deficiencies have on immunometabolism are needed to develop therapeutic methods of precision nutrition.

### 3.3. Thiamine

Thiamine pyrophosphate (TPP) is the main active form of thiamine (vitamin B1). Thiamine deficiency can occur post-BS in up to 49% of patients and cause nausea and constipation and eventually neurological and psychiatric complications including Wernicke-Korsakoff syndrome [47].

The intestines contain leukocytes as part of the gut-associated lymphoid tissue (GALT). Peyer's patch (PP) located in the submucosa of the small intestine contains immunocompetent cells, including B cells for the induction and regulation of the IgA response [48]. Naïve B cells in PP use thiamine for ATP synthesis via the TCA cycle. Once B cells differentiate into IgA-secreting plasma cells, there is a preference to use glycolysis for ATP production as well as the synthesis of the IgA antibody [49]. Research has shown that B cell immunometabolism maintenance is B1-mediated, specifically in PP sites because of its crucial role for IgA responses [50]. Thiamine deficiency can lead to regression of PP as well as decreased IgA antibody responses that protect against infections and maintain homeostasis with the microbiota [51].

While mammals cannot synthesize endogenous thiamine, the intestines are exposed to a dietary and bacterial source of thiamine [52, 53]. Most dietary thiamine exists as TPP that is hydrolyzed to free thiamine before absorbed by the small intestines, then transported to colonocytes via a carrier-mediated process involving thiamine transporter genes, *SLC19A2* (THTR-1) and *SLC19A3* (THTR-2) [54–56]. Thiamine produced from gut flora in the large intestine exists as both free and phosphorylated (TPP) forms [9, 57]. Microbiota-generated TPP can be absorbed into colonocytes efficiently that contributes to the host thiamine status [52, 55, 57]. Metagenomic analyses reveal that *Bacteroides fragilis* and *Prevotella copri* (Bacteroidetes); *Clostridium difficile*, some *Lactobacillus* spp., and *Ruminococcus lactaris* (Firmicutes); *Bifidobacterium* spp. (Actinobacteria); and *Fusobacterium varium* are vitamin B1 producers containing a thiamine biosynthesis pathway [49, 58]. Thiamine levels have been enhanced as a result of soy fermentation with *Streptococcus thermophilus, Lactobacillus helveticus, B. infantis*, or *B. longum* [59, 60]. In contrast, *Faecalibacterium* spp. (Firmicutes) is unable to synthesize thiamine in spite of requiring it for their own growth [58]. Having two sources of thiamine (dietary and bacterial) indicates competition for vitamin B1 between the host and certain intestinal bacteria.

### 3.4. Folate

Two forms of dietary folate exist: monoglutamate and polyglutamate. Folate deficiency is associated with elevated homocysteine, macrocytic anemia, leucopenia, and neural tube defects [47]. Folate abnormalities after RYGB increased as high as 29% of postoperative subjects [61]. Folate is also essential for DNA methylation, which can be altered by weight loss after BS, and may partly explain depletion of folate reserves postsurgically [62].

Folate helps maintain the immunological homeostasis in the intestines, specifically for the survival of regulatory T (Treg) cells that prevent excessive immune responses [49, 63]. Folate deficiency in mice resulted in reduction of Treg cells in the small intestine [64]. Mice fed with a folate-deficient diet showed a higher susceptibility to intestinal inflammation [65]. Folate deficiency can reduce proliferation of T lymphocytes that can cause abnormal nucleotide synthesis resulting in DNA damage. This reduction in T lymphocyte proliferation can also decrease resistance to infection in folate-deficient humans and animals [66, 67].

The human gut is exposed to a dietary and bacterial source of folate [56]. *Bacteroides fragilis* and *Prevotella copri* (Bacteroidetes); *Clostridium difficile, Lactobacillus plantarum, L. reuteri, L. delbrueckii* ssp. *bulgaricus*, and *Streptococcus thermophilus* (Firmicutes); some species in *Bifidobacterium* spp (Actinobacteria); *Fusobacterium varium* (Fusobacteria); and *Salmonella enterica* (Proteobacteria) can produce folate [49, 58]. Many probiotic strains such as *B. adolescentis* and *B. pseudocatenulatum*, *Lactobacillus plantarum*, *L. delbrueckii ssp. bulgaricus*, and *L. reuteri* can enhance folate production in the large intestine [49, 68–70]. However, further investigation is needed to determine if bacterial folate can significantly affect host folate status [9].

### 3.5. Vitamin B12

B12 deficiency can occur due to inadequate secretion of intrinsic factor and bypassing of the duodenum in malabsorptive bariatric procedures. A study involving RYGB subjects, B12 deficiency was observed in 33.3% postoperatively [34]. In patients who have had RYGB, most of the stomach and duodenum are bypassed that limits normal breakdown and binding to intrinsic factor (IF), making it the main reason for deficiency after surgery [3]. Deficiency of B12 can lead to deleterious consequences including anemia and neuropsychiatric symptoms such as numbness, memory disturbances, and dementia [47].

The microbiota express transporters that bind to corrinoids (analogues of B12) and use B12 as a cofactor for synthesis of odd-chain fatty acids, cholesterol, propionic acid, and branched-chain amino acids, used for the TCA cycle and generation of ATP. B12 requirement for the conversion of homocysteine to methionine can cause elevated homocysteine when B12 is insufficient [9]. B12 regulates gene expression in the gut, and over 80% of microbial species encode B12–dependent genes [9, 71]. B12 acts as a modulator for immune cells, especially CD8+ lymphocytes and natural killer (NK) cells [72–74]. B12 deficiency may increase IL-6 production and alter Treg cell counts in circulation [75]. Lambs fed with B12-deficient diets showed suppression of immunity that may be associated with proinflammatory responses [76].

Bacterial B12 biosynthesis is where adenosylcobalamin is synthesized from precorrin and absorbed by the large intestine [49, 58]. B12 is one of the least produced vitamins in the GM. By assessing the presence and absence of genome annotations, it is predicted that *Bacteroides fragilis* and *Prevotella copri* (Bacteroidetes), *Clostridium difficile*; *Faecalibacterium prausnitzii*, *Ruminococcus lactaris*, *and Lactobacillus reuteri* (Firmicutes); *Bifidobacterium animalis*, *B.infantis*, and *B.longum* (Actinobacteria); *Fusobacterium varium* (Fusobacteria); and *Pseudomonas denitrificans* (Proteobacteria) have functional roles in the B12 biosynthesis pathway [58, 77–82]. Furthermore, *Lactobacillus plantarum* and *L. coryniformis* from fermented food produce B12, and *Bifidobacterium animalis* produces B12 during milk fermentation [83, 84]. More research is needed to understand B12 export from bacterial cells [85].

### 3.6. Vitamin D

Metabolic bone disease is caused by persistent vitamin D (VitD) deficiency and linked to BS [86]. In 51 observational studies assessing vitD status in patients undergoing BS, the mean (25(OH)D) level was less than 30 ng/ml, (which is the minimum recommended level for optimal long-term health), before and after BS, despite various vitD supplementation regimens [87].

There are no vitD receptors (VDR) in prokaryotic cells, meaning that any effects of vitD on the microbiota would occur indirectly through the host that alter the microbiome [9]. VitD plays a critical role in the intestinal immune system and mucosal barrier function together with Vitamin A. The colonocytes can produce vitD; however, insufficient vitD can lead to decreased intestinal synthesis and possibly contribute to colon cancer and inflammatory bowel disease [88, 89]. VitD can modulate specific immune responses such as stimulating Th1 and Th2 cell proliferation that inhibits anti-inflammatory cytokines (IL-4 and IL-10) and induces proinflammatory cytokines (IL-1, TNF-*α*, IFN-*γ*) [90]. This type of immunomodulation may contribute to increased inflammation through production of IL-17 and IFN-*γ* in VDR–deficient mice [9, 91]. Other vitD-regulated immunity involves promoting Tregs and inhibiting B cell development and function. Genome-wide expression studies using a monocyte-derived dendritic cell model revealed that active vitD is pivotal in regulating immunometabolism such as the TCA cycle, oxidative phosphorylation, and ATP synthesis. These cells are influenced by vitD and can decrease their antibacterial activity and T cell immune response [92–95].

The GM enhances the expression of the VDR in intestinal epithelial cells. Products such as short chain fatty acids (SCFA), specifically butyrate, enhance cathelicidin formation in colonocytes that are regulated by vitD [96–98]. The human cathelicidin gene (*CAMP*) encodes for a specific antimicrobial peptide LL-37 in coloncytes, and activation of this cathelicidin is vitD-mediated [99, 100]. On the other side, VitD intake has an impact on microbial composition; however, research is conflicting with some data showing a negative association with *Prevotella* and a positive association with *Bacteroides* (Bacteroidetes) [101]. One cause for inconsistent results between studies is assessing vitD “dose” (e.g., sun exposure, dietary vitD intake, and serum 25(OH)D), which has led to a lack of knowledge regarding the effects of vitD on bacterial composition [95]. More research is needed to determine the benefits of vitD on the microbiota.

### 3.7. Iron

Iron deficiency, resulting in iron deficiency anemia, is the most common cause worldwide for anemia [102]. In individuals with obesity, the chronic inflammatory state related to obesity might be a possible risk factor for iron deficiency, which is also called the anemia of inflammation [103]. Both components make anemia highly complex, especially after BS [104]. Iron deficiency can be prevalent preoperatively and worsen after BS with a range of prevalence from 1 to 54% depending on the type of surgery [105]. The consequences of anemia after BS can be abnormal functions of tissues, such as blood, brain, and muscles, making prevention and treatment of iron deficiency imperative [105].

Enterocytes take up iron via metal transporters. Iron homeostasis is controlled by hepcidin, an antimicrobial peptide, that binds to and degrades the cellular iron exporter, ferroportin [106]. A regulatory interaction between iron and the immune system, called nutritional immunity, occurs when the host organism sequesters iron from pathogens as a defense mechanism [107]. Hepcidin inhibits iron transfer into circulation from the enterocytes, macrophages, and iron-storing hepatocytes, thereby starving pathogens of iron [107, 108]. Iron itself promotes lymphocyte and macrophage differentiation, antimicrobial immune effector function, and immune cell metabolism. The iron content of macrophages modulates their response to cytokines, such as IFN-*γ*, that are activated by NK and Th1 cells [109]. Mice fed with an iron-deficient diet exhibited suppression of the cellular immune response associated with impaired T-lymphocyte proliferation and IFN-*γ* secretion [110]. Iron deficiency can affect the nutrient metabolism as yeast grown in iron-poor environments that led to changes in glucose metabolism, amino acid biosynthesis, and lipid biosynthesis because of iron-dependent enzymes [111].

Iron availability significantly impacts the GM. Iron fortification has shown to lower *Bifidobacterium* but increase *Escherichia coli* [112]. During very low iron conditions, dysbiosis can occur with decreases in *Roseburia* spp./*Eubacterium rectale*, *Clostridium* Cluster IV members, and *Bacteroides* spp., while *Lactobacillus* spp. and *Enterobacteriaceae* increase along with a decrease in SCFA [113]. Iron supplementation may lead to a more pathogenic gut profile with an unfavorable ratio of *Enterobacteria* to *Bifidobacteria* and *Lactobacilli* due to certain pathogens needing iron to thrive unlike beneficial bacteria such as *Lactobacilli* [114]. Iron supplementation can adversely affect the microbiome by increasing prevalence of pathogens such as *Salmonella Clostridium difficile*, *Clostridium perfringens*, and *Escherichia coli,* leading to gut inflammation [115]. Several studies of colitis in animals suggest that oral iron supplementation could exacerbate intestinal inflammation, indicating that the parenteral iron administration may be a better treatment option for iron deficiency [116–120]. Additionally, intravenous iron replacement has been recommended after BS to minimize deficiency [121, 122]. There is a need to fully understand the balance between the iron metabolism and microbial population residing in the gut of BS patients.

### 3.8. Gut-Targeting Therapies

There is evidence that micronutrient deficiencies affect the microbiome and consequently host metabolism [9]. Recent data have identified “potentially beneficial microbes” as species found in genera *Bifidobacterium*, *Lactobacillus*, *Akkermansia*, *Fecalibacterium*, *Eubacterium*, *Roseburia*, *Ruminococcus*, and *Blautia* [123–126]. Studies have also reported certain bacterial species as “potentially detrimental microbes” including some from the genera *Clostridium*, *Enterobacter*, *Enterococcus*, *Bacteoides*, and *Ruminococcus* [124, 127–129]. Therapies such as prebiotics, probiotics, postbiotics, and functional foods that modulate the gut may be useful to restore microbial homeostasis and balance inflammation [130].  Table 2 illustrates studies published in the context of gut-targeting therapy research among BS subjects.

### 3.9. Prebiotics

Prebiotics are classified as “a selectively fermented ingredient that allows specific changes, both in the composition and/or activity in the gastrointestinal microflora that confers benefits upon host well-being and health” [131]. Nondigestible oligosaccharides and fructooligosaccharides (FOS) are prebiotics that can stimulate the growth of beneficial bacteria such as *Bifidobacteria*. There are common foods that contain FOS, i.e., garlic, onion, artichoke, and asparagus as well as commercially available supplements. Prebiotics can modulate the gut and can positively influence host health such as increased calcium, magnesium, and iron absorption and improved lipid profile [132]. Prebiotics are food substrates, such as plant complex polysaccharides, for probiotic microorganisms, explaining how diet can affect the microbiome [130]. Consumption of prebiotics is also associated with restoration of gut barrier integrity, enhanced absorption of micronutrients, and reduction of LPS, a toxic molecule found in gram-negative bacteria [133–137]. The role of diet in the gut with fast food consumption is associated with reduced microbial richness compared to high-fiber foods that are associated with a higher proportion of healthy microbes (Bacteroidetes), high levels of SCFAs (anti-inflammatory mediators), and a lower proportion of obesity-associated gut microbes (Firmicutes) [138]. There is evidence that prebiotics, such as inulin, promote beneficial *Bacteroides* operational taxonomic units but also stimulated harmful bacteria Firmicutes and some Proteobacteria [139].

A study showed the use of FOS for 15 days among BS patients that increased weight loss; however, the combination of FOS with a probiotic (synbiotic) failed to significantly lower inflammatory markers, although there was a reduction in their absolute values [140]. Synbiotics (a mixture of pre- and probiotics) are a good approach to benefit the host as they can selectively stimulate the growth and/or enhance the metabolism of certain health-promoting bacteria [132, 141, 142]. Additional studies are necessary to identify the specific prebiotics and their effects in the BS population.

### 3.10. Probiotics

Probiotics are “live microorganisms which when administered in adequate amounts confer a health benefit on the host” [143]. They are known to improve intestinal homeostasis by regulating microbial components and metabolites [144]. Certain probiotic species, that are commonly found in healthy intestines such as *Lactobacillus*, *Bifidobacterium*, and *Saccharomyces*, are used as a supplement to improve the health of the microbiota. Lactic acid bacterial (LAB) strains, specifically *L. lactis*, *L. Plantarum*, *L. reuteri*, and *L. acidophilus*, have shown to produce B vitamins, including folate and B12, from fermented food and indicate bioavailability to the host. Additionally, Bifidobacterial strains such as *B. bifidum*, *B. animalis*, and *B. longum* as well as *Streptococcus thermophilus* are also among probiotic species to improve B-vitamin levels in foods fermented with them [145]. Oral supplementation with probiotics, specifically *L. reuteri*, has been shown to increase circulating levels of vitD as well as the VDR expression and VDR activity in the host [146]. Probiotic strains *L. plantarum* and *rhamnosus* showed increases in the VDR expression and activity, which can lead to increases in the VDR target gene cathelicidin [147, 148].

Probiotics, especially *Lactobacillus* and *Bifidobacterium* species, can improve host health through protection of tissue integrity and reducing proinflammatory cytokine production such as TNF-*α* [149–151]. Probiotics are shown to interact with enterocytes and dendritic, Th1, Th2, and Treg cells in the intestines as an immunomodulatory effect involving proinflammatory and/or anti-inflammatory actions [152]. Probiotics can alter the GM profile to induce adaptive immune responses that protect the host from toxins and inhibit inflammation [153, 154]. GALT has shown to interact with probiotics like *L. bulgaricus* and *S. thermophilus* to mount an immune response known as adhesion that blocks pathogens from adhering to the host cell binding sites [155, 156]. Probiotic treatments should be carefully given to patients as the risk of infections and adverse effects, especially in inflammatory disease states, are not completely understood.

BS can cause malabsorption, changes in bile acid metabolism, gastric pH, and hormone secretion that in turn lead to GM changes. Research does indicate that BS has a positive impact on the microbiome as it correlates to metabolic parameters and weight loss. However, recent studies indicate that, although there are increases in MGR after BS, they still remain with low gene richness (as defined by metagenomic species signatures including high or low gene count classes), and the gut is absent of complete restoration even 5 years after surgery [157]. A study supplementing with Lactobacillus daily in post-BS subjects showed higher B12 levels as well as greater weight loss and reduction in bacterial overgrowth [158]. Additionally, RYGB patients given a probiotic supplement showed significant improvement in serum inflammatory markers including reduction in TNF-*α* as well as better weight loss [159]. Probiotics also have shown improvement in GI symptoms and quality of life as well as increased microbial diversity among BS patients [160, 161]. Further investigations are needed to determine the right probiotic or the combination of various strains and doses and the timing and supplementation period needed, as well as needs of consideration of individual's health status and disease type.

### 3.11. Postbiotics and Functional Foods

Identifying the molecules that are depleted in the gut after BS and then supplementing the diet with either the depleted molecule or a gut-signaling molecule that can be converted to the bioactive molecule is a novel approach that can encompass postbiotics. Postbiotics are nonviable bacterial products or metabolic byproducts from probiotic bacteria that can still benefit the host without administering associated risks with live microorganisms that can cause infection or adverse effects in inflammatory disease states. Research has shown that even bacteria supernatant can modulate the host immune response [130, 162]. Postbiotic intervention is an area of research that is showing potential to treat or prevent dysbiosis-driven diseases; however, much more research is needed to determine its usefulness in the BS population.

Functional foods like polyphenols, including flavanoids and omega-3 fats, can have beneficial roles beyond basic nutrition in the microbiota. Many foods and beverages that contain polyphenols, including vegetables, fruits, red wine, tea, and coffee, have antioxidant, antimicrobial, and anticarcinogenic as well as cardio- and neuroprotective effects [163]. Omega-3 fats, specifically from fish, may have protective effects against several disorders including cardiovascular, neurodegenerative, neuropsychiatric, inflammatory diseases, and some cancers [164, 165]. Studies have found that the prebiotic effects of polyphenol supplementation increased the abundance of *Bifidobacteria* and *Lactobacillus* and butyrate-producing bacteria as well as decreased Bacteroidetes that could be responsible for lowering metabolic syndrome markers [166, 167]. Flavanols have shown to increase *Bifidobacteria* and *Lactobacilli* and decrease clostridia counts with their potential prebiotic effects [168]. Omega-3 supplements have similar benefits with increases in *Bifidobacterium*, *Roseburia*, *Bacteroides*, *Prevotella*, and *Lactobacillus* as well as butyrate-producing bacteria, with decreases in *Faecalibacterium prausnitzii* and *Akkermansia* and Firmicutes/Bacteroidetes ratio, which could have beneficial effects on many health disorders [164, 169, 170]. These functional foods may benefit some populations. However, evidence using functional foods among BS patients is lacking, which warrants further investigation.

### 3.12. Nutritional Transcriptomics

The GM and its metabolites have a great impact on the nutrition and health status of the host. Fully understanding how genes influence the gut and how bacteria produce essential and modulatory metabolites that affect immune responses as well as nutrient metabolism can develop a healthy, symbiotic relationship between host and GM technological advances, specifically high-throughput metatranscriptomic investigations toward microbial expression profiling have revealed changes in the expression of metabolic pathways related to disease pathogenesis in the human microbiome. This allows for more individualized analysis of the gut during dietary interventions to reveal alterations in the microbial community gene expression profiles [171, 172]. Transcriptomics hold great potential to improve health; however, expression profiles of human GM are susceptible to great intra and intersubject variability [171].  Table 3 demonstrates studies using transcriptomic analysis among BS patients. Looking at changes in the gene expression of B12 pathway-encoding genes at post-RYGB surgery, Sala and colleagues found that RYGB affected several certain genes which may be associated with postoperative B12 deficiency [173]. However, no other studies involving transcriptomic methods in the context of micronutrient supplementation research were found among BS subjects. Other studies involving transcriptomic methods looked at effects on muscle proteome, weight loss, and subcutaneous fat in relation to insulin resistance [174–176]. These studies were included in Table 3 to depict the use of transcriptomics among BS subjects.

**Figure 1:**
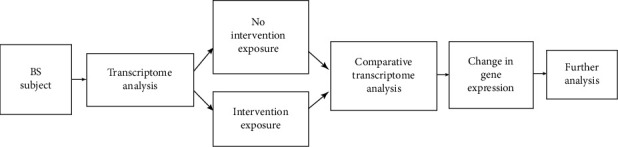
Schematic representation of an experimental format for nutritional studies using transcriptomic analysis.

Nutritional transcriptomics can use microarray analysis of samples from nutritionally relevant studies to identify many genes that are regulated at the mRNA level by exposure to different dietary interventions (i.e., micronutrient supplement and/or gut-targeting therapies) [177].  Figure 1 shows an adaptation to a study design using transcriptomic methods in nutrition research to analyze effects to immune and inflammatory processes.

Studies relating the effects of micronutrient supplementation on the gene expression in non-BS populations are shown in Table 4. Many studies used vitD supplementation with varying outcomes. Some showed an effect on the gene expression in subjects with obesity and among women [178, 179]. Other studies using vitD supplementation found no significant effects on the skeletal muscle transcriptome or on the concentration of certain cytokines among older adults [180, 181]. Studies that involved folic acid and B12 supplementation had effects on DNA methylation of genes, and vitamin A supplementation had a positive impact on the gene expression pattern of relevant cytokines [182, 183]. Although these gene-nutrient expressions may be sensitive biomarkers, they are limited by potential confounding effects that undermine their value. This could be resolved by developing markers based on expression profile “signatures” rather than one single gene. Identifying “signatures” and involving characteristic patterns of the differential gene expression could be used to look for biomarkers in cells that have been exposed to different levels of micronutrients [177].

## 4. Discussion

BS can have a positive impact on microbial diversity and gene richness of the gut, although complete restoration of the gut is not evident [157]. Micronutrients can promote growth of beneficial gut microbes, such as *Bifidobacteria*, *Akkermansia*, and *Lactobacilli*, and the effects of micronutrient deficiencies commonly occurring after BS can disturb the immune homeostasis in the gut [124]. Sufficient micronutrient levels are pivotal to maintaining the intestinal immune system and regulating immunometabolism. However, micronutrient supplementation studies show conflicting results and should be explored further to understand the appropriate therapeutic dose and route to achieve proper serum levels as well as GM composition after BS. Research does indicate a critical role of micronutrients in gut-related immune functions among non-BS populations [49]. Our review reveals the scarcity of literature that examines the role of micronutrients in modulating the GM among the BS population. This limitation should be considered when interpreting the findings in this review.

There is consistent, yet limited evidence that certain gut-targeting therapies may play a beneficial role in the microbiome for BS patients. Prebiotics and probiotics in the BS population may improve bacterial diversity and weight loss and decrease inflammatory markers. One study showed improvement in B12 levels. Although postbiotics and functional foods studies are not found in the BS community, they hold promise for health benefits by promoting positive gut modulation. Further research needs to be conducted in BS patients to understand the role they may play in gut health.

This review demonstrates the importance of understanding how interventions (i.e., micronutrient and gut-targeted therapies) affect and restore microbial homeostasis after BS to determine if they could further improve the barrier function and immune system of the intestinal tract, ultimately improving clinical outcomes. A highly individualized analysis of these interventions is needed to reveal the appropriate dose and route necessary to prevent and treat micronutrient deficiencies as well as fully restore the gut after BS. Nutritional transcriptomics shows great potential in providing this type of analysis. Data is lacking in this area with only one micronutrient supplementation study involving transcriptomic analysis among BS patients. Well-defined supplement intervention studies are needed that include a wide range of individuals with different types of BS to better understand the intra- and interindividual variability in the responsiveness of the individuals and their microbiomes to different supplement interventions.

**Figure 2:**
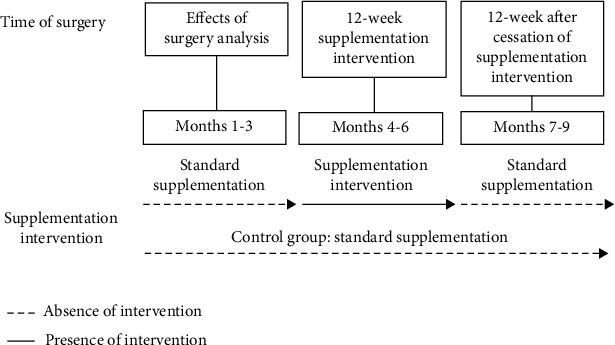
Supplementation intervention study design in BS subjects.

Approaches to consider for supplement intervention studies would incorporate defining the population, baseline “healthy” GM, using model systems (i.e., in vitro, in vivo, animal, and fermentation systems modeling specific portions or the entire GI tract), and well-designed human studies [184]. Defining the population should categorize subjects based on deficiency cause, additional health-concerns, types of BS, differences in the GM, and sociological considerations. The treatment effectiveness can be significantly altered by decreased intake or lack of absorption, which will differ according to surgery type. It is also important to consider subjects' habits (i.e., timing of eating, meal ordering, and how much exercise they are engaging in) when conducting intervention studies. Establishing a baseline “healthy” GM should consider the factors that define the population such as altered micronutrient requirements based on surgery type, age, gender, etc. These studies should use model systems to test the effectiveness of potential interventions considering the specifications of the defined population and “healthy” gut. This will help control for the intra- and intersubject variability in response to the same intervention. Translating this data to outcomes and improvements by conducting comparable, high quality clinical trials according to CONSORT guidelines is important in developing probiotics useful for humans [184, 185]. Intervention studies should utilize housekeeping genes that are suitable as reference genes and stability value [186]. A supplement intervention (i.e., micronutrients, probiotics, and functional foods) study design for BS subjects is illustrated in Figure 2.

Approaches designed to identify gene expression “signatures” for micronutrient research should be developed and tested widely in clinical research. Using the microarray expression profiling, from a cohort representative of the demographics including BS and different micronutrient deficiencies, will help establish these prognostic signatures. Defining gene expression signatures based on prediction of restoring micronutrient insufficiency as well as restoring the gut in BS patients is important. This will involve analyzing those that have BS and receive an intervention that restores their nutrient levels or their GM composition to establish a mechanistic link between a gene expression signature and adequate nutrition and MGR. Gene expression technologies to help predict risk and treatment benefit in nutritional deficiencies or low MGR show a potential value for those who have BS [177].

Concerns and limitations involved in nutritional transcriptomics in human studies include (1) time-consuming and resource-intensive work, (2) lack of access to target tissues, (3) technical challenges of isolating sufficient high quality RNA from tissues, and (4) controlling for interindividual variation in human subjects [177]. The utility and sensible application of this approach can improve the limitations. For example, by using plasma or urine analysis for specific metabolites can be considered as biomarkers of specific dietary intake [187, 188]. Performing a controlled study with the adequate sample size and peripheral blood mononuclear cells as target tissues might decrease these limitations; however, this is not a miracle technique [188–191]. When applied correctly, it can be powerful to detect small differences in the gene expression induced by supplementation (i.e., micronutrient, probiotics, and functional foods) to generate data that contributes to understanding the responder and nonresponder phenomenon as well as how to fully restore gut health after BS.

## 5. Conclusions

BS is an effective treatment for severe obesity; however, micronutrient deficiencies can occur. Although people receive the same obesity treatment, there can be major variations in interindividual responses. Understanding the mechanisms and factors involved in this phenomenon is critical. GM plays a role in vitamin synthesis, mineral absorption, and immunometabolism. Further investigations are needed to determine whether GM changes following BS could be correlated with micronutrient deficiencies and adverse medical outcomes. Personalized recommendations for micronutrient supplementation among BS patients have not been evaluated, and the role that the gut might play represents a potential benefit in this research area. Nutritional transcriptomic analysis in clinical trials could help understand nutritional deficiencies, poor responders, and improving clinical responses after BS.

## Figures and Tables

**Table 1:** Common bacterial species and their role in human health.

Phylum	Genus	Species	Role in health and disease	Reference
Acinobacteria	*Bifidobacterium*	*B. bifidum*, *B. animalis and B. longum*, *B. infantis*, *B. lactis, B. adolescentis*, *B. pseudocatenulatum*	(i) Members of this genus have been investigated to play a beneficial role in diarrhea, colorectal cancer, and inflammatory bowel disease. (ii) They have common binding sites on epithelial cell and prevent gastrointestinal infections by competitive exclusion.	[11, 12]
Firmicutes	*Clostridium*, *Faecalibacterium*, *Lactobacillus*, *Staphylococcus*, *Streptococcus*, *Ruminococcus*, *Roseburiam*, *Enterococcus*	*C. difficile*, *F. prausnitzii*, *L. reuteri*, *L. plantarum*, *L. delbrueckii*, *L. bulgaricus*, *L. coryniformis*, *L. acidophilus*, *L. rhamnosus*, *L. paracasei*, *L. helveticus*, *Streptococcus thermophilus*, *Ruminococcus lactaris*, *Ruminococcus bromii*	(i) High concentrations of *Lactobacillus* spp. are observed in the microbiota of individuals with obesity causing dysbiosis. (ii) Members of genus *Lactobacillus* can enhance the epithelial barrier, promote mucus adhesion, provide protection against invasion of pathogens, and produce antimicrobial substances such as bacteriocins.	[11, 13]
Bacteriodetes	*Bacteroides*, *Prevotella*	*B. fragilis*, *P. copri*	(i) Members of genus *Bacteroides* are involved in the carbohydrate metabolism. (ii) They affect the immune function through development of the gut-associated lymphoid tissue (GALT) and mature immune system, aid in production of antimicrobial molecules, prevent colonization by pathogens in the gastrointestinal tract, and play a role in proper development of immune tolerance. (i) Members of genus *Prevotella* have shown to improve glucose metabolism, produce healthy short chain fatty acids, and have anti-inflammatory effects. (ii) Some studies show the association of *Prevotella* species with inflammatory conditions, glucose intolerance, and insulin resistance.	[11, 14, 15]
Proteobacteria	*Salmonella*	*S. enterica*	(i) Members of this genus are intestinal pathogens implicated in gastroenteritis and typhoid fever.	[11, 16]
Fusobacteria	*Fusobacteria*	*F. varium*	(i) Members of this genus are associated with the increased risk of pancreatic and colorectal cancers and involved in pathological conditions such as Crohn's disease (CD) and ulcerative colitis (UC).	[11, 17]
Verrucomicrobia	*Akkermansia*	*A. muciniphila*	(i) This species is associated with intestinal health and metabolic status improvement in type 2 diabetes and obese subjects. (ii) It demonstrates the ability to strengthen impaired gut barrier by adhering to the intestinal epithelium and enhance enterocyte monolayer integrity. (iii) Compared to healthy individuals, *A. municiphila* is fewer in CD and UC patients and is thus associated with gut health.	[11, 18]

**Table 2:** Studies showing use of gut-targeting therapies/interventions in the bariatric surgery population.

Reference	Study Objective	Study Design	Sample	Intervention	Duration	Main results found
[158]	To study the effects of the probiotic administration on bacterial overgrowth, quality of life, gastrointestinal (GI) symptoms, and weight loss after surgery.	Prospective randomized controlled trial	35 RYGB patients. Control group (*n* = 20) Probiotic group (*n* = 15)	Supplementation with 2.4 billion colonies of Lactobacillus daily postoperatively	6 months	(1) Statistically significant reduction in bacterial overgrowth. (2) Higher postoperative B12 levels (3) Greater percent excess weight loss
[159]	To investigate the effect of probiotic supplementation on inflammatory factors, anthropometric indices, and vitamin B12, folate, homocysteine, and 25-hydroxy vitamin D3 levels in One Anastomosis Gastric Bypass-Mini Gastric Bypass (OAGB-MGB) surgery patients.	Placebo-controlled, double-blind, randomized clinical trial	46 women candidates for (OAGB-MGB) surgery. Placebo group (*n* = 23) Probiotics group (*n* = 23)	Probiotic supplement (Familact®) containing seven species of probiotic bacteria (Lactobacillus casei, Lactobacillus rhamnosus, Streptococcus thermophilus, Bifidobacterium breve, Lactobacillus acidophilus, Bifidobacterium longum, Lactobacillus bulgaricus)	Total 16 weeks (4 weeks before surgery to 12 weeks after surgery)	(1) Significant improvement in serum inflammatory markers including reduction in TNF-*α* in the probiotic group. (2) Percent weight loss and decreased BMI in the probiotic group. (3) No significant difference in serum levels of vitamin B12, folate, and homocysteine between the placebo and probiotic groups
[140]	To study the effects prebiotic and synbiotic supplementation on inflammatory markers and anthropometric indices	Randomized, triple-blind, placebo-controlled study	9 patients undergoing open RYGB surgery and 9 healthy individuals	3 groups: 6 g/d of placebo (maltodextrin), prebiotic (fructo-oligosaccharide, FOS), or synbiotic (FOS+Lactobacillus and Bifidobacteria strains)	15 days	(1) Increased weight loss and BMI reduction in the placebo and prebiotic groups. (2) No significant changes in inflammatory markers between groups.
[160]	To investigate the effects of probiotic supplementation on improvement of symptomatic GI symptoms after surgery	Double-blind, prospective, randomized trial	60 patients who underwent RYGB divided into 3 groups of 20 subjects each.	3 groups: probiotic group A—1 g Clostridium butyricum MIYAIRI, Probiotic group B—Bifidobacterium longum BB536 and digestive enzymes group	2 weeks	Improvement in GI symptoms and quality of life in all the 3 groups after the intervention period.
[161]	To study the comparative effect of probiotics and placebo on hepatic, inflammatory, and clinical outcomes postlaparoscopic sleeve gastrectomy (LSG).	Randomized, double-blind, placebo-controlled, trial	100 morbidly obese subjects with nonalcoholic fatty liver disease who underwent LSG.	2 groups: probiotic (2 capsules per day of Bio-25 Supherb) group (*n* = 50) and placebo group (*n* = 50)	6 months	Microbiota diversity increased in both the groups after 6 months of surgery and decreased at 12 months after surgery. No improvements were seen on hepatic, inflammatory, and clinical outcomes.

**Table 3:** Studies showing the use of transcriptomic analysis in bariatric surgery populations.

Reference	Study aim	Population characteristics	Methods	Study outcomes
[173]	To study changes in gene expression levels of B12 vitamin pathway-encoding genes post-RYGB surgery.	20 obese women with adult-onset type 2 diabetes undergoing RYGB surgery.	Serial gastrointestinal biopsies were collected from subjects before and 3 months after surgery. Affymetrix Human GeneChip 1.0 ST microarray was used to assess gene expression levels. Real-time quantitative PCR (RT–qPCR) was used to validate the findings.	RYGB affected several pathway-encoding genes which may be associated with postoperative B12 deficiency. Significant changes included increased cubilin and decreased transcobalamin 1 levels.
[174]	To investigate the effect of obesity and RYGB surgery on the human skeletal muscle proteome.	7 obese female subjects undergoing RYGB and 4 lean females as control subjects.	Basal muscle biopsies were obtained before and 3 months after RYGB surgery. Quantitative mass spectrometry and microarray analyses were performed on protein and RNA isolated from the muscle biopsies.	RYGB surgery had significant effects on the skeletal muscle proteome. 2,877 quantifiable proteins were identified by proteomic analysis amongst which 395 proteins were altered before surgery, and 280 proteins differed significantly postsurgery. 49 proteins returned to normal levels after surgery.
[175]	To evaluate the effect of diet and surgery induced weight loss on DNA methylation and hydroxymethylation levels.	Control group—9 normal weight women, energy-restricted Mediterranean-based dietary treatment group—22 obese women, and bariatric surgery group—14 obese women.	Anthropometric and 12-hour fasting blood sample was collected before and after 6 months of intervention from all subjects. Assessments done included lipid and glucose biomarkers, global hydroxymethylation (by ELISA), LINE-1, SERPINE-1, and IL-6 (by MS-HRM) methylation levels.	SERPINE-1 methylation and weight loss responses were associated. Increased IL-6 methylation levels after diet induced weight loss and decreased levels of the same after bariatric surgery. DNA methylation differed as per obesity treatment and may serve as a biomarker for obesity.
[176]	To study changes in the gene expression in the subcutaneous adipose tissue after RYGB based on high/low insulin resistance (IR) state.	4 morbidly obese women with high IR and 4 morbidly obese women with low IR.	Microarray analysis was used to assess subcutaneous adipose tissue samples before and 2 years after RYGB surgery.	Shared and exclusive groups of differentially expressed genes (DEG) are found in both high and low IR subjects. In high IR group, the downexpressed DEG is related to transcription regulation as well as several pathways including cytokine-cytokine receptor interaction, cancer, chemokine signalling, etc.

**Table 4:** Studies related to effects of micronutrient supplementation on the gene expression in nonbariatric surgery populations.

Reference	Study aim	Population description	Intervention	Methods	Main findings
[180]	To study the effect of vitamin D supplementation on the whole genome gene expression in the skeletal muscle.	Vitamin D deficient frail older adults. Calcifediol supplementation group (*n* = 9) & placebo group (*n* = 12)	10 *μ*g per day calcifediol for 6 months	Affymetrix HuGene 2.1ST arrays were used for the whole genome gene expression profiling of muscle biopsies obtained before and after 6 months of intervention for all subjects.	No significant effect of supplementation was seen on the skeletal muscle transcriptome of frail older adults.
[178]	To study the effect of vitamin D supplementation on transcriptome.	Obese subjects (*n* = 18) and normal weight subjects (*n* = 18) each randomized into supplementation and placebo groups.	50 *μ*g (2000 IU) daily dose of vitamin D for 12 weeks	Data were collected at baseline, 6 and 12 weeks from all subjects. The peripheral blood gene expression was analysed using GlobinLock oligonucleotides followed by RNA sequencing.	Vitamin D supplementation affected the gene expression in obese subjects but not in normal weight subjects.
[182]	To identify long-term supplementation effects of folic acid and vitamin B12 on genome wide DNA methylation.	87 subjects with mildly elevated homocysteine levels (*n* = 44 folic acid and B12 group, *n* = 43 placebo group)	400 *μ*g folic acid and 500 *μ*g vitamin B12 per day for 2 years	Infinium HumanMethylation450 BeadChip was used for genome-wide DNA methylation, and DNA samples were collected before and after intervention from all subjects.	Long-term folic acid and B12 supplementation have effects on DNA methylation of genes including those implicated in the developmental processes.
[181]	To study the effect of vitamin D supplementation on the gene expression and plasma cytokine levels.	305 community-dwelling individuals aged 65 years and above	2 treatment groups with 4000 IU & 2000 IU vitamin D3 supplementation, respectively, for 12 months and one placebo group.	Genome-wide genotypes were measure at baseline, and transcriptome and plasma cytokine levels were measured at baseline and after 12 months of intervention.	No significant effect of high dose vitamin D supplementation was observed on the gene expression and concentration of selected cytokine levels.
[179]	To analyze the effects of vitamin D supplementation on the gene expression	Vitamin D supplementation group (*n* = 47) and placebo group (*n* = 47)	20,000 IU dose of vitamin D per week for 3 to 5 years	Blood samples were drawn for preparation of RNA, and microarray analysis was used to determine the mRNA gene expression in the blood.	Between the two groups, no significant changes in the gene expression were found after supplementation. On analyzing separately based on gender, women showed significant changes on the gene expression. In total, 99 genes were found to be regulated.
[183]	To determine the effects of vitamin A supplementation on gene expression cytokines secreted by TCD4+ lymphocytes.	Vitamin A supplementation group of atherosclerotic patients (*n* = 16), atherosclerotic patients receiving placebo group (*n* = 15), healthy subjects receiving vitamin A supplementation (*n* = 12)	25000 IU retinyl palmitate per day for 4 months.	Fasting blood samples were collected before and after 4 months from all subjects. Gene expression pattern of relevant cytokines of CD4+ T cells including was determined by real-time PCR.	Reduced gene expression of IFN-*γ* and T-bet in all patients after supplementation. Increased gene expression of IL-4 in subjects who received supplementation. Positive role of vitamin A supplementation on the gene expression.
